# *Nocardia ignorata* Infection in Heart Transplant Patient

**DOI:** 10.3201/eid2611.202756

**Published:** 2020-11

**Authors:** Victoria A. Muggia, Yoram A. Puius

**Affiliations:** Albert Einstein College of Medicine, Bronx, NY, USA; Montefiore Medical Center, Bronx

**Keywords:** bacteria, heart transplant, Nocardia ignorata, transplant infectious diseases

**To the Editor:** We read with interest the recent description of pulmonary *Nocardia ignorata* infection (*1*). We report a similar infection in an orthotopic heart transplant recipient, which likely began as a pulmonary infection with dissemination to soft tissue, without known exposure. Risk factors included tacrolimus, steroids, older age, and posttransplant intensive care unit admission (*2*).

The patient was a 66-year-old African American man with a history of ischemic cardiomyopathy. After implantation of a left ventricular assist device, infectious complications included *Enterococcus faecalis* device infection and extended spectrum β-lactamase–producing (ESBL) *Klebsiella* urosepsis. The course after left ventricular assist device explantation and orthotopic heart transplant was complicated by tamponade requiring a pericardial window and an ESBL *Klebsiella* urinary tract infection treated with meropenem. Because of leukopenia, *Pneumocystis* prophylaxis was changed from trimethoprim/sulfamethoxazole to atovaquone 2 weeks posttransplant. ESBL *Klebsiella* bacteremia recurred 6 weeks later, again treated with meropenem. 

The patient returned 6 months posttransplant with 10 days of cough and dyspnea. Chest computed tomography demonstrated bilateral nodules with cavitation, bronchiectasis, and spiculation. We initially treated the patient with meropenem and doxycycline. Results from severe acute respiratory syndrome coronavirus 2 swab test, respiratory pathogen panel, fungal studies, and sputum culture were nondiagnostic. We obtained no additional pulmonary samples. 

Due to severe left calf pain, venous duplex was performed, revealing a nonvascular mass. The patient reported no trauma, soil contact, or recent travel. The abscess was aspirated, demonstrating branching gram-positive beaded rods. The isolate was identified by a reference laboratory (Mycobacteria and Nocardia Laboratory, University of Texas Health Center at Tyler, Tyler, TX, USA) by partial 16S rRNA sequencing as a 99.51% match with *Nocardia ignorata*, with susceptibilities identical to the isolate in Rahdar et al. (*1*). Brain magnetic resonance imaging results were unremarkable. The patient’s respiratory status and leg pain quickly improved and he was discharged on long-term trimethoprim/sulfamethoxazole and doxycycline. Because of renal insufficiency, trimethoprim/sulfamethoxazole was switched to moxifloxacin after 2 weeks. Chest radiograph results were improving 3 months later. 
